# Associations among Cognitive Functions, Plasma DNA, and Diffusion Tensor Image along the Perivascular Space (DTI-ALPS) in Patients with Parkinson's Disease

**DOI:** 10.1155/2021/4034509

**Published:** 2021-02-16

**Authors:** Hsiu-Ling Chen, Pei-Chin Chen, Cheng-Hsien Lu, Nai-Wen Tsai, Chiun-Chieh Yu, Kun-Hsien Chou, Yun-Ru Lai, Toshiaki Taoka, Wei-Che Lin

**Affiliations:** ^1^Department of Diagnostic Radiology, Kaohsiung Chang Gung Memorial Hospital and Chang Gung University College of Medicine, Kaohsiung, Taiwan; ^2^Department of Neurology, Kaohsiung Chang Gung Memorial Hospital and Chang Gung University College of Medicine, Kaohsiung, Taiwan; ^3^Brain Research Center, National Yang-Ming University, Taipei, Taiwan; ^4^Institute of Neuroscience, National Yang-Ming University, Taipei, Taiwan; ^5^Department of Radiology, Nagoya University Graduate School of Medicine, Japan

## Abstract

**Background:**

Parkinson's disease (PD) is a common neurodegenerative disease associated with accumulation of misfolding proteins and increased neuroinflammation, which may further impair the glymphatic system. The purpose of this study was to utilize diffusion tensor image analysis along the perivascular space (DTI-ALPS) to evaluate glymphatic system activity and its relationship with systemic oxidative stress status in PD patients.

**Methods:**

Magnetic resonance imaging and neuropsychological tests were conducted on 25 PD patients with normal cognition (PDN), 25 PD patients with mild cognitive impairment (PD-MCI), 38 PD patients with dementia (PDD), and 47 normal controls (NC). Oxidative stress status was assessed by plasma DNA level. Differences in ALPS-index among the subgroups were assessed and further correlated with cognitive functions and plasma DNA levels.

**Results:**

The PD-MCI and PDD groups showed significantly lower ALPS-index compared to normal controls. The ALPS-index was inversely correlated with plasma nuclear DNA, mitochondrial DNA levels, and cognitive scores.

**Conclusions:**

Lower diffusivity along the perivascular space, represented by lower ALPS-index, indicates impairment of the glymphatic system in PD patients. The correlation between elevated plasma nuclear DNA levels and lower ALPS-index supports the notion that PD patients may exhibit increased oxidative stress associated with glymphatic system microstructural alterations.

## 1. Introduction

Parkinson's disease (PD) is a common neurodegenerative disease presenting motor and nonmotor symptoms. Several coexisting misfolding-associated proteins play important roles in the pathogenesis of PD, including cortical and limbic Lewy bodies [1], neurofibrillar tangles, and senile plaques [2]. Abnormal increased accumulation of misfolding proteins, together with blood-brain barrier (BBB) disruption and neuroinflammation, is related in a vicious cycle and might highly associate with the development of PD and subsequent disease progression [3]. Elucidation of the relationship between brain waste clearance routes and central nervous system (CNS) oxidative stress status may assist with disease monitoring and may potentially delay clinical progression.

**Figure 1:**
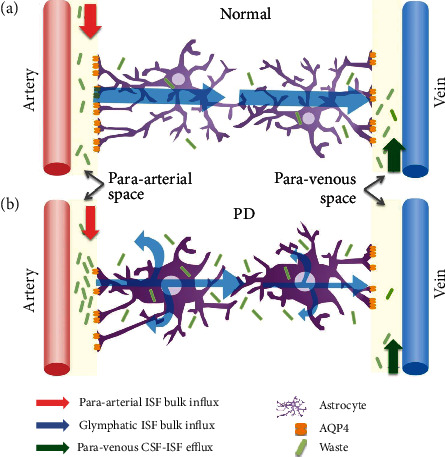
Illustration of the brain waste clearance between the perivascular space and glymphatic system. The glymphatic system is a waste drainage system in which cerebrospinal fluid (CSF) in the para-arterial space exchanges with interstitial fluid (ISF) with the aid of aquaporin-4 (AQP4) water channels on the astroglial endfeet and is drained into the perivascular space, from which it eventually reaches the lymphatic nodes. The system is driven by para-arterial ISF bulk influx (red arrow), glymphatic ISF bulk influx (blue arrow), and para-venous CSF-ISF efflux (green arrow). In disease status, such as neurodegeneration disorder, neuroinflammation in interaction with systemic oxidative stress can alter the function of BBB and astrocytes and waste clearance efficiency in the glymphatic system.

The glymphatic system is a recently discovered waste drainage system in the brain which involves movement of cerebrospinal fluid (CSF) along the perivascular space to promote elimination of soluble proteins, including misfolding proteins [4]. In this system, CSF is exchanged with interstitial fluid (ISF), facilitated by the aquaporin-4 (AQP4) water channels in the astroglial endfeet/endfeet of astrocytes. ISF is drained into the perivascular space, eventually reaching the lymphatic nodes [4]. Preservation of normal glymphatic system function, particularly of astrocyte cells, is therefore essential for maintenance of the clearance system (Figure 1(a)).

In PD patients, neuroinflammation interacting with systemic oxidative stress may induce further CNS reactive astrogliosis [5], potentially impairing the glymphatic system. In addition, aging, a primary risk factor of PD, has been found to associate with decreased glymphatic activity and could be attributed to increase reactive gliosis. Accumulation of waste materials may render the brain more vulnerable to development of a degenerative pathology or perhaps enhance the progression of cognitive dysfunction (Figure 1(b)). Therefore, investigation of glymphatic system integrity and its association with peripheral oxidative stress level differences and cognitive decline in PD patients is required.

Activity of the glymphatic system has indeed been studied by intrathecally administered tracers in animal experiments [4]. More recently, MRI has been applied to noninvasively evaluate the brain glymphatic system [6]. By using diffusion tensor image analysis along the perivascular space (DTI-ALPS), Taoka et al. found decreased glymphatic activity in patients with Alzheimer's disease and mild cognitive impairment [6]. The lower water diffusivity along the perivascular space was related to Alzheimer's disease severity, demonstrating the effectiveness of the DTI-APLS method for evaluation of glymphatic system activity.

The purpose of the present study was to utilize DTI-ALPS, by acquiring an index for diffusivity along the perivascular space (ALPS-index), to evaluate glymphatic system activity in PD patients. We analyzed various degrees of glymphatic system activity by performing diffusion tensor studies of normal control subjects and PD patients, further divided into three PD subgroups. Furthermore, we measured the plasma DNA level to indicate the systemic oxidative stress status in all participants. Correlations among ALPS-index, cognitive function, and systemic oxidative stress were analyzed to reveal possible mechanisms.

## 2. Materials and Methods

### 2.1. Participants

The study protocol was approved by the Chang Gung Memorial Hospital's Institutional Review Committee in Human Research. All participants or their caregivers were provided written informed consent prior to participation. Eighty-eight PD patients were prospectively enrolled in the study at the Neurology Department of Kaohsiung Chang Gung Memorial Hospital. Patients were included if they had a definitive diagnosis of idiopathic PD [7]. The disease severity and functional status of each patient were evaluated with the Unified Parkinson Disease Rating Scale (UPDRS) [8], the modified Hoehn and Yahr stages (HY stage) [9], and the Schwab and England Activities of Daily Living Scale (SE-ADL) [10].

PD patients were further separated into three subgroups, classified as PD patients with normal cognition (PDN), PD patients with mild cognitive impairment (PD-MCI), and PD patients with dementia (PDD) in accordance with the Movement Disorder Society Task Force Guidelines [11]. Cognitive impairment was defined as a score 1.5 SD below the normative mean in each of the domains [11]. PDN was defined as less than two domains of cognitive impairment. PD-MCI was defined as one score at −1.5 SD in each of two or more domains but without dementia. PDD was defined as impairment in more than one cognitive domain with a Mini-Mental State Examination (MMSE) score of less than 26 [12]. In addition, forty-seven healthy subjects without neurological disease, psychiatric illness, alcohol or substance abuse, or head injury were recruited from the hospital as the normal control (NC) group.

### 2.2. Evaluation of Cognitive Function

Neuropsychological evaluations of five cognitive domains, attention and working memory, executive, language, memory, and visuospatial, were conducted using subtests from the Cognitive Ability Screening Instrument (CASI) [13] and the Wechsler Adult Intelligence Scale-III [14]. The Mini-Mental State Examination (MMSE) [12] was also performed to assess the general cognitive function of the subjects.

### 2.3. Laboratory Examinations of Plasma DNA in Peripheral Circulation

Plasma levels of nuclear and mitochondrial DNA were assessed in all study participants. The blood was drawn by venipuncture at the forearm on the same day as the MRI study and the neuropsychological testing. The plasma DNA was measured by a real-time quantitative polymerase chain reaction (RT-PCR) assay (Roche LightCycler, Roche, Grenzach-Wyhlen, Germany) for the *β*-globin gene (present in all nucleated cells) and ND2 genes (specific for mitochondrial DNA). Quantitative results were expressed as ng/ml. Procedural details are described in a previous study [15].

### 2.4. DTI-ALPS-Index Acquisition

The MRI data were acquired on a 3.0-T MRI scanner (SIGNA, General Electric Healthcare, Milwaukee, WI, USA). The subject's head was immobilized with foam pillows inside the coil to diminish motion artifacts. DTI scans were obtained using a single-shot echo planar imaging sequence. Images were acquired axially parallel to the anterior-posterior commissure (AC-PC) to cover the entire brain with the following parameters: repetition time (TR)/echo time (TE) = 15800/77 ms; field of view (FOV) = 25.6 cm; matrix size = 128 × 128; voxel size = 2 × 2 × 2.5 mm^3^; number of excitations (NEX) = 3, 55 slices without gap; *b*value = 1000 s/mm^2^; and 13 noncollinear directions and one nondiffusion-weighted T2 image.

**Figure 2:**
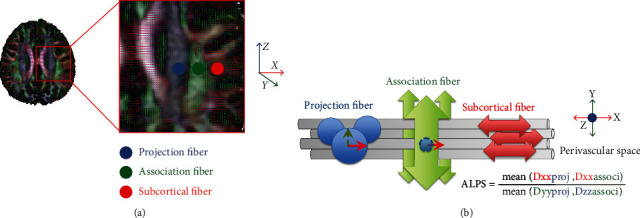
(a) DTI color map shows the direction of the projection fibers (blue; *z*-axis), association fibers (green; *y*-axis), and the subcortical fibers (red; *x*-axis). Three ROIs are placed to measure diffusivities of the three fibers. (b) Schematic diagram presents the relationship between the direction of the perivascular space (gray cylinders), the fibers, and the formula of the ALPS-index. Note that the direction of the perivascular space is perpendicular to both projection and association fibers.

We used the dTV. II.13 k + software (Dept. Biomedical Information Sciences, Graduate School of Information Sciences, Hiroshima City University) [16] to calculate the diffusion metric images. The DTI-ALPS method has been detailed in a previous report [6]. The software estimates a diffusion tensor at each voxel of the brain and outputs a series of images including a color-coded fractional anisotropy (FA), mean diffusivity (MD), and diffusivity maps in the direction of the *x*-, *y*-, and *z*-axis (Dxx, Dyy, Dzz). It is used to evaluate the diffusivity along the direction of the perivascular space by comparing with those of projection fibers and association fibers on a slice at the level of the lateral ventricle body (Figure 2(a)). At that level, the direction of the perivascular space is perpendicular to the ventricle wall and is thus mostly in the right-left direction (*x*-axis) on the axial plane. The direction is also perpendicular to the direction of both the projection fibers (mostly in the *z*-axis) and the association fibers (mostly in the *y*-axis) (Figure 2(b)). Thus, the diffusivity along the *x*-axis at regions with projection/association fibers will at least partially represent the diffusivity along the perivascular space. On a color-coded FA map of the plane at the level of the lateral ventricle body, we placed a 5 mm diameter spherical region of interest (ROI) in the area of the projection fibers (blue on Figure 2(a)), the area of the association fibers (green on Figure 2(a)), and the area of the subcortical fibers (red on Figure 2(a)) in the left hemisphere. In this study, we obtained measurements only in the left hemisphere, as all subjects were right-handed and the SLF is thick enough to place the ROI on the left side.

Further, we calculated an index which we will call as ALPS-index in order to evaluate the activity of the glymphatic system in individual cases. This index is provided by the ratio of two sets of diffusivity value which are perpendicular to dominant fibers in the tissue, that is the ratio of mean of *x*-axis diffusivity in the area of projection fiber (Dxproj) and *x*-axis diffusivity in the area of association fibers (Dxassoc) to the mean of *y*-axis diffusivity in the area of projection fiber (Dyproj) and *z*-axis diffusivity in the area of association fibers (Dzaccoc) as follows.

(1) $$\begin{array}{c} \text{ALPS index}=\text{mean}\frac{\left(\text{Dxxproj},\text{Dxxassoci}\right)}{\text{mean}}\left(\text{Dyyproj},\text{Dzassoci}\right). \end{array}$$

In the area of projection fibers, the dominant fibers run in the direction of *z*-axis, and both *x*-axis and *y*-axis are perpendicular to the dominant fibers. Similarly, in the area of association fibers, the dominant fibers run in the direction of *y*-axis, and both *x*-axis and *z*-axis are perpendicular to the dominant fibers. The major difference for the water molecule behavior between *x*-axis diffusivity in both area (Dxproj and Dxassoc) and the diffusivity which is perpendicular to them (Dyproj and Dzassoc) would be the existence of the perivascular space. The ALPS-index measures the diffusivity from the comportment along the perivascular space direction in those perpendicular projection fibers and association fibers to represent the integrity of the glymphatic system.

### 2.5. Statistical Analyses

The age data were compared by analysis of variance (ANOVA). The sex data were compared using the Pearson chi-squared test. The disease severity, MMSE, CASI, neuropsychological test scores, and plasma DNA levels were analyzed using the analysis of covariance (ANCOVA) model with the participant's age and sex as covariates. The statistical significance was set at *p* < 0.05.

#### 2.5.1. Analysis of ALPS-Index Differences between NC and PD Subgroups

The ANCOVA was conducted with age and sex as covariates to compare the ALPS-index between the NC, PDN, PD-MCI, and PDD groups. Post hoc tests with Bonferroni correction were performed to investigate which pairs of groups differed after an overall difference had been established. The PD patients were divided into 2 subgroups according to their modified HY stage at the same time: (1) early PD:HY≦2 and (2) late PD:HY > 2, to perform the quantitative analysis of the ALPS-index.

#### 2.5.2. Correlations among ALPS-Index, Cognition Scores, Plasma DNA Levels, and Disease Severity

After confirming data normality, correlations were measured using Spearman's correlation coefficient to evaluate the relationships among the ALPS-index, plasma DNA levels, and neuropsychological test scores of study subjects. In the PD subgroups, similar analyses were also conducted to evaluate the relationship between the ALPS-index and clinical disease severity. All statistical thresholds were set at *p* < 0.05 (SPSS V.17, Chicago, IL, USA).

## 3. Results

### 3.1. Clinical Characteristics and Cognitive Profiles among Groups

The demographic data are summarized in Table 1. Using ANOVA analysis, it was determined that age data (*p* = 0.034) differed significantly among the groups, and the PDD group was significantly older than the control group in the post hoc analysis. There were no significant differences in sex, disease duration, and disease severity (UPDRS, modified HY stage, SE-ADL) between the NC group and the overall PD group.

The neuropsychological results are demonstrated in Table 2. The PDD group had significantly lower CASI and MMSE scores than the other groups. On the neuropsychological assessments, there was no significant difference between the NC and the PDN groups. The PDD group had significantly lower attention, executive, memory, speech and language, and visuospatial function scores than the NC and PDN groups. Additionally, the PDD group exhibited poorer performance than the PD-MCI group in aspects of attention (digit span, attention, orientation), executive (digit symbol coding, similarity, matrix reasoning, abstract thinking), memory (short-term memory, long-term memory, information), speech and language (vocabulary, comprehension, language), and visuospatial function (picture completion, drawing). The PD-MCI group also had poorer cognitive function than the NC and PDN groups in many aspects of functions including attention (digit span), executive (digit symbol coding, similarity, arithmetic, matrix reasoning), speech and language (vocabulary, comprehension), and visuospatial function (picture completion, block design).

### 3.2. Plasma DNA Level among Groups

We observed significantly higher plasma nuclear DNA levels in the PDD group compared with the NC, PDN, and PD-MCI groups (Table 1); however, there was no intergroup difference between the NC, PDN, and PD-MCI groups. No significant plasma mitochondrial DNA difference was detected among all subgroups.

### 3.3. ALPS-Index Differences among Groups

**Figure 3:**
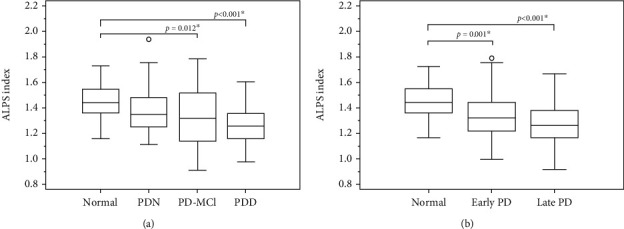
Quantitative analysis of the ALPS-index in the PDN, PD-MCI, PDD patient groups (a), and in the early PD and late PD patient groups (b) in comparison with the healthy control group.

Compared with the NC group, the PD-MCI and PDD groups exhibited significantly lower DTI-ALPS-index scores (*p* = 0.012 and *p* < 0.001, respectively). There was no significant difference between the PD subgroups (Figure 3(a)). The NC group also revealed significant higher ALPS-index scores compared to early PD (*p* = 0.001) and late PD (*p* < 0.001) subgroups. There was no significant difference between early and late PD subgroups (*p* = 0.346) (Figure 3(b)).

### 3.4. Correlations of ALPS-Index with Plasma DNA Level, Cognitive Function, and Disease Severity

**Figure 4:**
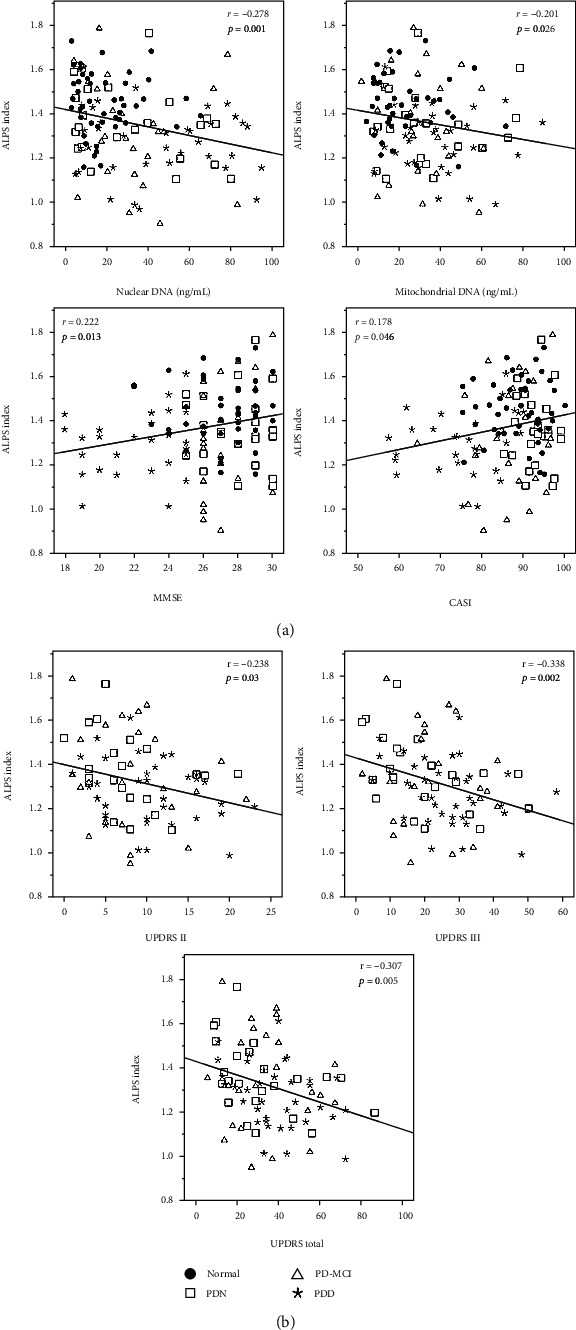
Correlation among the ALPS-index, plasma DNA, and cognitive function in the NC and PD group (a) and in the PD subgroups (b).

Among all groups, there were significant negative correlations between the DTI-ALPS-index and nuclear DNA level (*r* = −0.278, *p* = 0.001), mitochondrial DNA level (*r* = −0.201, *p* = 0.026), and positive correlations with MMSE (*r* = 0.222, *p* = 0.013) and CASI (*r* = 0.178, *p* = 0.046) (Figure 4(a)). In the PD subgroups, the ALPS-index scores were negatively correlated with the UPDRS II (*r* = −0.238, *p* = 0.03), UPDRS III (*r* = −0.331, *p* = 0.002), and UPDRS total score (*r* = −0.307, *p* = 0.005) (Figure 4(b)).

## 4. Discussion

This study revealed novel evidence indicating that alterations in ALPS-index are associated with systemic oxidative stress during PD cognition decline and severe disease stage. Investigating associations between inflammation biomarkers, glymphatic system changes, and clinical status in PD patients may facilitate earlier diagnosis and improve prognostic accuracy.

Firstly, PD-MCI and PDD patient groups exhibited lower ALPS-index compared to NC. Meanwhile, plasma nuclear DNA levels were higher in the PDD group compared to the NC, PDN, and PD-MCI groups; however, no significant difference was found among the NC, PDN, and PD-MCI groups. Secondly, a lower ALPS-index was associated with elevated disease severity and inferior MMSE and CASI scores. Thirdly, our results corroborate the hypothesis that systemic oxidative stress plays a significant pathogenic role in diffusivity along the perivascular space. Of note, we discovered that the ALPS-index may be a cardinal marker of characteristic brain glymphatic system alterations in various stages of PD.

### 4.1. ALPS-Index and the Glymphatic System

The anatomical profile of the recently discovered glymphatic system is complex; it has been defined as a para-arterial CSF influx pathway and para-venous clearance routes, which are functionally coupled by interstitial bulk flow supported by astrocytic AQP4 water channels [4]. In the periventricular region, abundances of medullary arteries and veins accompanying the perivascular space run in a right-left direction (*x*-axis) and are the major drainage pathway of the glymphatic system. By measuring the diffusivity from the comportment along the perivascular space direction in those perpendicular projection fibers and association fibers (Figure 2), the ALPS-index might represent the integrity of the glymphatic system on a slice at the level of the lateral ventricle body [6]. Taoka et al. has reported a decreased ALPS-index in patients with mild cognitive impairment and Alzheimer's disease [6]. The lower diffusivity may result from long-term exposure to A*β* in the subarachnoid CSF, subsequently suppressing glymphatic transport [17]. Similarly, we herein demonstrated a decreased ALPS-index in the PD-MCI and PDD groups compared with the NC group. When the ratio is close to 1, it means that the influence of the water diffusion along the perivascular space is minimal, and a larger ratio will represent larger water diffusivity along the perivascular space. In addition, significant positive correlations between disease severity, MMSE, and CASI scores and the ALPS-index further support the effectiveness of the ALPS-index in representing impairment of the glymphatic system. Even though the relationship between plasma nuclear DNA and the ALPS-index is relatively divergent, this might be due to the fact that the plasma nuclear DNA is not affected by the ALPS-index alone. Besides, the ALPS-index is not sensitive enough to detect abnormal perivascular diffusivity differences between early PDN and NC cases; moreover, there may be other concurrent factors which impair cognitive performance in PD-MCI and PDD cases.

### 4.2. Possible Pathophysiology of Altered Glymphatic System and Oxidative Stress

There is at present a dearth of research utilizing neuroimaging to investigate the relationship between systemic inflammation and the glymphatic system in PD patients. Increased plasma DNA level may support the existence of a critical primary pathophysiology of PD, specifically abnormal systemic oxidative stress and the acceleration of peripheral immune cell infiltration into the brain. Systemic inflammation involving various pathways such as BBB dysfunction and the infiltration of peripheral immune cells and circulating cytokines have been widely reported in PD [18–20]. Of note, BBB disruption and inflammation are actually connected in a vicious cycle. Our previous study indeed demonstrated the elevation of plasma nuclear DNA in PD which can even occur in early onset PD patients [15]. In addition, higher peripheral apoptotic lymphocytes may be associated with a decreased cognition-associated functional network in PD patients with cognitive deficit [5]. Meanwhile, impaired BBB may attract more mast cells and further release of inflammatory mediators, subsequently leading to BBB disruption. The present study may provide indirect evidence of peripheral inflammation related with PD degeneration and cognitive impairment in patients.

Increased systemic oxidative stress may also lead to malfunction of the glymphatic system by decreasing the convective flow, CSF-to-ISF turnover, resulting in impaired waste clearance [21]. Defensins are peptides which are released as part of the immune response to defend the brain against pathogens [22]. On the abluminal side of the perivascular space, abnormal defensin expression has been demonstrated to activate T cells, viruses, and inflammatory mediators which enter the brain, thereby hindering the glymphatic flow. Inadequate defensin expression also leads to downregulation of LRP1 and decreased A*β* clearance. Meanwhile, astrocytes play a key role in the relationship between BBB and the glymphatic system, with endfeet enveloping the capillary walls, allowing transport of nutrients between endothelial cells and the parenchyma [23]. Systemic toxic substances increasingly enter the brain, possibly triggering excitotoxicity and neuronal death [24]. In addition, neuroinflammation has also been reported in astrocytes and neurodegeneration in PD [25, 26], possibly compromising the AQP4 water channels in astrocytes and impeding the convective flow of the glymphatic system [4]. As observed in this study, the impaired glymphatic system with decreased ALPS-index may be representative of increased neuronal loss associated with disease progression and cognitive function deterioration in PD patients.

Recent evidences have proposed that the glymphatic system is more active (90%) during sleep [27] to establish a state to efficiently clear the CNS of the metabolic waste produced throughout the wakeful period [28]. The regulation of glymphatic activity is likely influenced by the differences between wakeful and sleep states as well as the homeostatic mechanism [28], which are affected in PD, leading to the development of sleep disorders, such as insomnia and daytime sleepiness [29]. It is highly likely that the glymphatic system plays a central role in the relationship between sleep disturbances and PD; therefore, further investigating the glymphatic system and its role in the development and progression of these conditions is of significant importance.

### 4.3. Limitations

Notwithstanding the novel findings of this study, the results should be interpreted in light of some limitations. Although the ALPS-index measured at the periventricular region may represent focal glymphatic system integrity, global evaluation of the regional perivascular diffusivity is impossible due to the fact that ALPS was designed to particularly measure the DTI index in the periventricular area only. Another limitation is that the ROI was placed manually, which may be a subjective factor of our measurement. However, we tried to place ROI as objectively as possible.

According to Braak staging, the periventricular region is usually affected in late stage PD, thus resulting in an insensitivity to the use of ALPS in the evaluation of early PD. Degradation of white matter integrity or white matter hyperintensity is considered a contributor to cognitive decline in PD. Our manual assessment may not be able to totally eliminate those confounding effects. The factors associated with the systemic plasma oxidative stress are widely variable and difficult to be clarified in the present study. Elucidating the potential relationships between the ALPS-index and plasma biomarkers was also hindered by the cross-sectional design of this study. Future longitudinal studies may provide further insight into the vulnerability of distinct brain regions, as well as their degradation trajectories over the course of PD.

## 5. Conclusions

Lower diffusivity along the perivascular space, represented as lower ALPS-index, suggests impairment of the glymphatic system in PD patients. The results of this study demonstrate significant correlations between the diffusivity along the perivascular space, the MMSE, the CASI score, and disease severity, indicating that impaired water diffusivity is indeed related to PD severity. The association between elevated plasma nuclear DNA levels and lower ALPS-index supports the notion that PD patients may exhibit increased oxidative stress with possibly associated neuronal damage or death, consequently leading to extensive glymphatic system microstructural changes which are visible via DTI.

## Figures and Tables

**Table 1:** Demographic characteristics of the PD patients and normal controls.

	Normal group	Patients with PD (*n* = 88)			*p* value	
	(*n* = 47)	PDN (*n* = 25)	PD-MCI (*n* = 25)	PDD (*n* = 38)		
Sex (male, female)	15, 32	15, 10	8, 17	11, 27	0.056	
Age (years)	61.53 ± 4.75	60.08 ± 10.07	63.8 ± 8.70	65.76 ± 8.00	0.034	^∗^¶
Disease duration (years)	—	2.49 ± 1.91	3.07 ± 2.67	3.64 ± 4.28	0.333	
UPDRS I	—	3.16 ± 2.29	3.76 ± 3.00	3.76 ± 3.07	0.886	
UPDRS II	—	8.40 ± 6.31	9.48 ± 7.50	11.55 ± 8.17	0.262	
UPDRS III	—	19.56 ± 13.14	24.76 ± 15.41	27.47 ± 14.47	0.157	
UPDRS total	—	31.12 ± 20.26	38.00 ± 24.25	42.79 ± 23.98	0.213	
Modified HY stage	—	1.86 ± 1.25	2.16 ± 1.26	1.88 ± 0.84	0.418	
SE-ADL	—	83.6 ± 20.99	82.00 ± 22.55	85.26 ± 11.32	0.731	

CASI	88.76 ± 6.47	92.87 ± 3.80	86.62 ± 7.79	69.62 ± 15.66	<0.001	^∗^¶&†
MMSE	27.21 ± 2.06	27.92 ± 1.78	27.08 ± 1.32	20.00 ± 4.53	<0.001	^∗^¶&†

*Plasma DNA*						
Nuclear DNA (ng/mL)	19.34 ± 14.76	31.35 ± 26.18	32.03 ± 21.40	46.13 ± 33.67	<0.001	^∗^¶&†
Mitochondrial DNA (ng/mL)	31.54 ± 33.86	39.89 ± 31.04	45.36 ± 38.70	43.04 ± 31.17	0.206	

Data are presented as mean ± standard deviation. The age data were compared by analysis of variance (ANOVA). Sex data were compared by Pearson chi-squared test. Clinical severity score and plasma DNA data were compared by analysis of covariance (ANCOVA) after controlling for age and sex with Bonferroni correction. *p* value represented the comparison amounts of the PDN, PD-MCI, and PDD patients and the normal control group. ^∗^Significant differences among groups; ¶Significant differences between NC and PDD; &Significant differences between PDN and PDD; †Significant differences between PD-MCI and PDD.

**Table 2:** Neuro-psycological results of the PD patients and normal controls.

	Normal group	Patients with PD (*n* = 88)			*p* value	
	(*n* = 47)	PDN (*n* = 25)	PD-MCI (*n* = 25)	PDD (*n* = 38)		
*Attention function*						
Digit span	10.64 ± 2.68	11.76 ± 2.50	8.72 ± 2.46	7.11 ± 2.88	<0.001	^∗^§¶#&†
Attention	7.36 ± 0.94	7.68 ± 0.56	7.08 ± 1.04	5.87 ± 1.34	<0.001	^∗^¶&†
Orientation	17.64 ± 0.82	17.68 ± 1.41	17.24 ± 1.45	14.84 ± 4.27	<0.001	^∗^¶&†
*Executive function*						
Digit symbol coding	10.21 ± 3.46	10.36 ± 2.93	8 ± 2.25	5 ± 2.17	<0.001	^∗^§¶#&†
Similarity	9.64 ± 2.91	11.08 ± 2.53	8.04 ± 3.02	6.68 ± 2.45	<0.001	^∗^§¶#&†
Arithmetic	10.11 ± 3.17	10.56 ± 2.52	7.48 ± 2.06	6.74 ± 1.54	<0.001	^∗^§¶#&
Picture arrangement	9.47 ± 3.69	10.32 ± 2.63	8.4 ± 2.84	6.88 ± 2.37	<0.001	^∗^¶&
Matrix reasoning	9.09 ± 3.23	10.96 ± 2.84	8.68 ± 2.48	6.29 ± 2.25	<0.001	^∗^¶#&†
Abstract thinking	9.26 ± 1.85	9.92 ± 1.18	8.72 ± 2.01	6.74 ± 2.27	<0.001	^∗^¶&†
*Memory function*						
Short-term memory	9.12 ± 1.96	10.25 ± 1.64	9.15 ± 2.98	5.77 ± 2.90	<0.001	^∗^¶&†
Long-term memory	9.87 ± 0.49	10.00 ± 0.00	9.52 ± 1.045	8.79 ± 2.11	0.005	^∗^¶&†
Information	9.72 ± 3.11	10.48 ± 2.35	9.04 ± 2.41	6.92 ± 1.89	<0.001	^∗^¶&†
*Speech and language*						
Vocabulary	10.45 ± 3.35	11.84 ± 2.95	8.64 ± 3.03	6.32 ± 2.32	<0.001	^∗^§¶#&†
Comprehension	10.57 ± 3.20	11.24 ± 2.50	8.56 ± 3.01	6.25 ± 2.27	<0.001	^∗^§¶#&†
Language	9.83 ± 0.94	9.90 ± 0.29	9.60 ± 0.99	8.33 ± 1.39	<0.001	^∗^¶&†
*Visuospatial function*						
Picture completion	9.36 ± 3.14	10.24 ± 2.39	8.28 ± 2.56	6.24 ± 2.47	<0.001	^∗^¶#&†
Block design	9.26 ± 3.14	10.16 ± 2.72	7.00 ± 2.24	6.18 ± 2.61	<0.001	^∗^§¶#&
Drawing	9.68 ± 1.12	9.76 ± 0.60	9.84 ± 0.47	8.07 ± 2.41	<0.001	^∗^¶&†

Data are presented as mean ± standard deviation. The cognitive function data were compared by analysis of covariance (ANCOVA) after controlling for age and sex with Bonferroni correction. *p* value represented the comparison amounts of the PDN, PD-MCI, and PDD patients and the normal control group. ^∗^Significant differences among groups; §Significant differences between NC and PD-MCI; ¶Significant differences between NC and PDD; #Significant differences between PDN and PD-MCI; &Significant differences between PDN and PDD; †Significant differences between PD-MCI and PDD.

## Data Availability

The datasets generated and analyzed during the current study are not publicly available due to the restriction from the ethics review board but are available from the corresponding author on reasonable request.

## Abbreviations

AQP4: Aquaporin-4
BBB: Blood-brain barrier
CASI: Cognitive ability screening instrument
CSF: Cerebrospinal fluid
DTI-ALPS: Diffusion tensor image analysis along the perivascular space
ISF: Interstitial fluid
Modified H & Y scale: Modified Hoehn and Yahr staging scale
MMSE: Mini-Mental State Examination
NC: Normal control
PD: Parkinson's disease
PDN: PD with normal cognition
PD-MCI: PD with mild cognitive impairment
PDD: PD with dementia
UPDRS: Unified Parkinson's Disease Rating Scale
S & E scale: Schwab and England Activities of Daily Living Scale.
